# Accuracies of *Leuconostoc *phenotypic identification: a comparison of API systems and conventional phenotypic assays

**DOI:** 10.1186/1471-2334-7-69

**Published:** 2007-07-02

**Authors:** Wanla Kulwichit, Sumanee Nilgate, Tanittha Chatsuwan, Sunisa Krajiw, Chudaachhara Unhasuta, Anan Chongthaleong

**Affiliations:** 1 Department of Medicine, Faculty of Medicine, Chulalongkorn University, Bangkok, Thailand; 2 Department of Microbiology, Faculty of Medicine, Chulalongkorn University, Bangkok, Thailand

## Abstract

**Background:**

Commercial diagnostics are commonly used to identify gram-positive bacteria. Errors have been reported mostly at the species level. We have found certain phenotypic criteria used in API systems which significantly misidentify *Leuconostoc*, an emerging human pathogen, at the genus level. We also attempt to find practical, conventional phenotypic assays for accurate identification of this group of bacteria.

**Methods:**

Clinical isolates of catalase-negative, gram-positive coccoid or coccobacillary bacteria with non-β hemolysis in our institute during 1997–2004 were subject to an identification aid by API 20 STREP, following the instruction manual, as an aid to conventional phenotypic tests. Those identified as *Leuconostoc *by API 20 STREP were re-examined by the same kit and also by API 50 CHL according to the instruction manuals, by our *Leuconostoc *conventional phenotypic assays, by *Leuconostoc*- and *Lactobacillus*-specific PCR's, and, where possible, by 16S rDNA sequence analysis. In addition, catalase-negative gram-positive isolates during 2005–2006 which were resistant to vancomycin at high levels were also evaluated by the same phenotypic and genotypic assays.

**Results:**

Out of several thousands of clinical gram-positive isolates, 26 catalase negative gram-positive isolates initially identified as *Leuconostoc *by API 20 STREP and 7 vancomycin-resistant gram-positive catalase-negative bacteria entered the study. 11 out of the 26 isolates and all the 7 isolates were identified as *Leuconostoc *by API 20 STREP. Only 5 isolates, however, were confirmed by both genotypic and all defined conventional phenotypic criteria. API 50 CHL also failed to reliably provide accurate identification of *Leuconostoc*. We have identified key problem tests in API 20 STREP leading to misidentification of the bacteria. A simple, conventional set of phenotypic tests for *Leuconostoc *identification is proposed.

**Conclusion:**

The current API systems cannot accurately identify *Leuconostoc*. Identification of vancomycin-resistant, catalase-negative gram-positive bacteria should be performed by a few practical phenotypic assays, with assistance of genotypic assays where available.

## Background

*Leuconostoc *is a gram-positive coccoid or coccobacillary emerging human pathogen found in environment, foods and food products [1]. Risk factors of infection include antibiotic pressure, foreign device, or underlying immune defects. The organism is naturally highly resistant to vancomycin with MIC ≥ 256 μg/ml but could be successfully treated with penicillin with MIC ranging from 0.25 to 1.0 unit/ml. Commercial diagnostics are commonly used to identify gram-positive bacteria, with errors mostly at the species level [2,3]. Here we report inaccuracies of the Analytical Profile Index systems (API 20 STREP and API 50 CHL, Biomérieux, Inc., Lyon, France) in identifying *Leuconostoc *at the genus level. We also propose practical methods for clinical bacteriology laboratories to identify this organism.

## Methods

Clinical isolates of catalase negative gram-positive coccoid or coccobacillary pairs and chains with α- or γ-hemolysis in our institute during 1997–2004 were subject to an identification aid by API 20 STREP (bioMérieux, Inc., Lyon, France), following the instruction manual. Those identified as *Leuconostoc *by API 20 STREP were re-examined by the same kit and by API 50 CHL (bioMérieux, Inc., Lyon, France) according to the instruction manuals, by *Leuconostoc *conventional phenotypic assays, by *Leuconostoc*- and *Lactobacillus*-specific PCR's, and by 16S rDNA sequence analysis as previously described [4]. The 800-bp 16S rDNA fragment corresponds to Escherichia coli positions 10 to 806. The sequencing results were compared with those available in the GenBank, using BLASTN. Criteria for our conventional phenotypic assays for *Leuconostoc *are catalase-negative gram-positive coccoid or coccobacillary bacteria evaluated after growth in thioglycolate broth at 35°C for 24–48 hours [5], vancomycin MIC ≥ 256 μg/ml by Etest (AB BIODISK, Solna, Sweden), CO2 production from glucose in de Man, Sharp, Rogosa (MRS) broth (Difco, Detroit, MI, USA) with Durham tubes and negative pyrrolidonyl arylamidase (PYR), leucine arylamidase (LAP), and arginine dihydrolase (ADH) [6]. *Leuconostoc*-specific PCR was performed on all isolates as described [7], with slight primer modifications, as stated below. These modifications were to make the primer sequences most complementary and specific to *Leuconostoc *strains in GenBank. Forward and reverse primer sequences were 5'-CACAGCGAAAGGTGCTTGCAC-3' and 5'-GATCCATCTCTAGGTGACGCC-3', respectively. To further assess accuracy of the API 20 STREP kit, additional catalase-negative gram-positive coccoid or coccobacillary isolates during 2005–2006 with vancomycin MIC ≥ 256 μg/ml were also evaluated by the same phenotypic and genotypic assays (isolates 31–38 in Table 1). Our gold standard for *Leuconostoc *identification is that the organisms fulfill both conventional phenotypic criteria and either or both of the genotypic assays (PCR and 16S rDNA sequence analysis). As we suspected that some isolates might have been *Lactobacillus*, also a lactic acid bacteria with overlapping phenotypes, *Lactobacillus*-specific PCR was also performed on all isolates as described [8]. PCR using universal primers targeting bacterial 16S rRNA conserved sequences was also performed to ensure template quality. The forward primer Y1 corresponds to positions 20 to 43 in the *E. coli *16S rRNA sequence and the reverse primer Y2 corresponds to *E. coli *positions 361 to 338 [9]; this protocol gave positive results for all isolates in the study. *Leuconostoc mesenteroides *ATCC 8293, *Pediococcus pentosaceus *ATCC 33316, *Lactobacillus pentosus *ATCC 8041 and *Lactobacillus plantarum *ATCC 14917 served as controls for all assays. This study has been approved by The Institutional Review Board of The Faculty of Medicine, Chulalongkorn University.

**Table 1:** Comparison of various identification methods for 4 ATCC reference strains of *Lactobacillus*, *Pediococcus*, *Leuconostoc*, and 26 catalase-negative, gram-positive clinical isolates from 1997–2004 (numbers 1–26) initially identified as *Leuconostoc *by API 20 STREP or 7 isolates from 2005–2006 expressing high levels of vancomycin resistance (numbers 31–33 and 35–38).

**Isolate #-specimen type**	**API 20 STREP (% identity)**	**API 50 CHL (% identity)**	**Gram's staining results**	**ADH**	**LAP**	**MRS**	**PYR**	**Van**	**PCR for *Lactobacillus* and *Leuconostoc***	**Sequencing of 16S rDNA gene (% identity)**
*Lactobacillus plantarum* ATCC 14917*	*Enterococcus avium* (63.2)	*Lactobacillus plantarum* (99.9)	B	-/-	+/+	-	-/-	R	+ve *Lactobacillus*	*Lactobacillus plantarum* (100)

*Lactobacillus pentosus* ATCC 8041*	*Leuconostoc *(81.1)	*Lactococcus lactis *ssp *lactis* 1 (82.5)	B	-/-	+/+	+	-/-	R	+ve *Lactobacillus*	N/A

*Pediococcus pentosaceus* ATCC 33316*	*Leuconostoc* (39.3)	*Lactobacillus pentosus* (84.3)	C	+/+	+/+	-	-/-	R	Neg	*Pediococcus pentosaceus* (99.4)

*Leuconostoc mesenteroides* ATCC 8293	*Leuconostoc* (96.8)	*Leuconostoc mesenteroides *ssp *mesenteroides/dextranicum* 1 (95.7)	Cb	-/-	-/-	+	-/-	R	+ve *Leuconostoc*	*Leuconostoc mesenteroides* (99.3)

1-pus*	*Streptococcus suis* biotype I (85.6)	*Lactobacillus acidophilus* (97.4)	C-Ch	-/+^#^	+/-^#^	-	-/-	S	Neg	*Streptococcus suis* (100)

2-blood*	*Leuconostoc* (99.8)	*Lactococcus lactis *ssp *lactis* 1 (90.5)	Cb	+/+	-/-	+	-/-	R	Neg	N/A

3-corneal discharge	*Leuconostoc *(97.9)	*Lactobacillus brevis* 3 (98.8)	C-Ch	-/-	-/-	+	-/-	R	+ve *Leuconostoc*	N/A

4-ascitic fluid	*Leuconostoc *(99.9)	*Leuconostoc mesenteroides *spp *mesenteroides/dextranicum* 2 (99.9)	C	-/-	-/-	+	-/-	R	+ve *Leuconostoc*	*Leuconostoc lactis* or* garlicum* (99.5)

5-ascitic fluid*	*Leuconostoc *(93.6)	*Lactobacillus acidophilus* 1 (85.1)	Cb	+/+	-/-	+	-/-	R	Neg	N/A

6-blood	*Leuconostoc* (95.4)	*Leuconostoc lactis* (96.0)	Cb	-/-	-/-	+	-/-	R	+ve *Leuconostoc*	*Leuconostoc lactis* or *garlicum* (99.6)

7-blood	*Leuconostoc* (68.5)	*Leuconostoc lactis* (92.0)	Cb	-/-	-/-	+	-/-	R	Neg	N/A

8-blood*	*Streptococcus mitis* 1 (81.1)	*Lactobacillus acidophilus *(92.1)	Cb	-/-	+/-^#^	-	-/-	S	Neg	N/A

9-blood*	*Leuconostoc* (97.2)	*Leuconostoc mesenteroides* spp *cremoris *(99.9)	Cb	+/+	-/-	+	-/-	R	Neg	*Weissella cibaria* (100)

10-blood*	*Abiotrophia adiacens* (46.9) *Aerococcus viridans *2 (27.5)	*Lactobacillus delbrueckii *spp *delbrueckii* (78.1)	Cb	-/-	-/-	-	-/-	S	Neg	N/A

11-blood*	*Leuconostoc* (92.2)	*Lactobacillus acidophilus* (72.5)	C-Ch	-/-	-/+^#^	-	-/-	S	Neg	N/A

12-ascitic fluid*	*Lactococcus lactis* spp *cremoris* (47.2) *Leuconostoc *(45.1)	*Lactobacillus salivarius* (99.9)	C-Ch	-/-	-/-	-	-/-	R	+ve *Lactobacillus*	N/A

13-blood*	*Streptococcus sanguis* (49.2) other streptococci (48.5)	*Leuconostoc mesenteroides* spp *cremoris* (98.7)	Cb	-/-	+/-^#^	-	-/-	S	Neg	N/A

14-blood*	*Leuconostoc* (39.0) *Lactococcus lactis *ssp *cremoris *(37.9)	*Lactobacillus acidophilus* 1 (88.2)	C	-/-	-/+^#^	-	-/-	S	Neg	*Streptococcus pasteurianus* (99.9)

15-blood*	*Streptococcus bovis* (64.8)	*Leuconostoc lactis *(87.9)	Cb	-/-	+/-^#^	-	-/-	R	Neg	*Weissella confusa* (99.9)

16-blood*	*Enterococcus faecium* (98.7)	*Lactobacillus plantarum* 1 (98.6)	Cb	+/+	+/-^#^	-	+/-^#^	S	Neg	*Enterococcus faecium* (99.9)

17-blood*	*Aerococcus viridans* (62.6)	*Lactobacillus delbrueckii *spp *delbruekii* (95.5)	Cb	-/-	-/-	-	-/-	S	Neg	*Actinomyces odontolyticus* (98.9)

18-brain abscess*	*Streptococcus constellatus* (99.9)	*Lactobacillus acidophilus* (82.1)	C-Ch	+/+	+/-^#^	+	-/-	S	Neg	*Streptococcus anginosus* or *constellatus* (99.7)

19-blood*	*Streptococcus bovis* biotype II (64.8)	*Lactobacillus acidophilus *1 (98.1)	C-Ch	-/-	+/-^#^	+	-/-	S	Neg	*Streptococcus constellatus* (99.7)

20-blood*	*Leuconostoc *(92.2)	*Weissella viridescens *(99.8)	C	-/+^#^	-/+^#^	-	-/-	R	Neg	*Weissella viridescens *(99.9)

21-lung swab*	*Leuconostoc *(99.9)	*Lactobacillus acidophilus *(78.4)	Cb	+/+	-/-	+	-/-	R	Neg	*Weissella cibaria *(100)

22-bone*	*Leuconostoc *(99.6)	*Lactobacillus coprophilus* (96.9)	Cb	+/+	-/-	+	-/-	R	Neg	*Weissella confusa *(99.61)

23-NR*	*Leuconostoc *(98.8)	*Lactobacillus coprophilus* (96.9)	Cb	+/+	-/+^#^	-	-/+^#^	R	Neg	*Weissella confusa *(100)

24-NR*	*Aerococcus viridans* (48.5) *Lactococcus lactis *ssp *cremoris* (41.6)	*Leuconostoc mesenteroides *spp *cremoris* (94.7)	C-Ch	-/+^#^	+/-^#^	-	-/-	S	Neg	*Streptococcus anginosus* (99.9)

25-pus*	*Streptococcus constellatus (*99.9)	*Lactobacillus delbrueckii *(80.4)	C-Ch	+/+	+/-^#^	-	-/-	S	Neg	*Streptococcus constellatus* (99.7)

26-duodenal* content	*Leuconostoc *(89.9)	*Lactobacillus salivarius (*99.9)	Cb	-/-	+/-^#^	-	-/-	R	+ve *Lactobacillus*	*Lactobacillus salivarius* (100)

31-urine*	*Leuconostoc *(99.7)	*Lactobacillus acidophilus *1 (49.5)	Cb	+/+	-/-	+	-/-	R	Neg	*Weissella cibaria *(99.5)

32-gastric content	*Leuconostoc *(99.4)	*Leuconostoc lactis* (95.3)	C-Ch	-/-	-/-	+	-/-	R	+ve *Leuconostoc*	*Leuconostoc garlicum *or *lactis *(99.2)

33-tissue biopsy	*Leuconostoc* (99.9)	*Leuconostoc mesenteroides* spp *mesenteroides/dextranicum*2 (99.9)	C-Ch	-/-	-/-	+	-/-	R	+ve *Leuconostoc*	*Leuconostoc garlicum *or *lactis* (99.6)

35-ascitic fluid*	*Leuconostoc *(65.8)	*Pediococcus pentosaceus *1 (63.6)	C	-/-	+/+	-	-/-	R	neg	*Pediococcus stilesii* (91.5)

36-blood	*Leuconostoc *(92.8)	*Lactobacillus collinoides* or *fermentum* 1 (98.3)	Cb	-/-	-/-	+	-/-	R	neg	*Weissella confusa* (99.9)

37-pleural fluid*	*Leuconostoc* (69.2)	*Lactobacillus salivarius* (99.9)	Cb	-/-	+/+	-	-/-	R	neg	N/A

38-tissue biopsy*	*Leuconostoc *(82.7)	*Pediococcus pentosaceus* (99.9)	C	+/+	+/+	-	-/-	R	neg	*Pediococcus pentosaceus* (98.2)

ADH = arginine dihydrolase, LAP = leucine arylamidase, MRS = gas production in MRS broth, PYR = pyrrolidonyl arylamidase test, Van = vancomycin MIC; S = MIC in the range of 0.5–1.0 μg/ml; R = MIC ≥ 256 μg/ml NR = no record available, N/A = result not available. ADH, LAP, and PYR test results listed are from API 20 STREP and from conventional phenotypic assays, respectively. Discordant results between the two methods are marked with #. In Gram's staining results column, C = cocci, Cb = coccobacillary form, C-Ch = cocci in chain, B = bacilli. * = phenotype(s) opposite to what is (are) expected for *Leuconostoc *in our criteria above. API 20 STREP identifies *Leuconostoc *at the genus level only. PCR results are indicated as positive for *Leuconostoc *or *Lactobacillus *or negative for both protocols. 16S rDNA sequencing results are indicated together with % similarity to the closest GenBank sequences.

## Results

Our clinical bacteriology laboratory has a busy service, serving a 1,500-bed university hospital. Out of several thousands of gram-positive bacteria isolated during 1997–2004, 26 catalase-negative gram-positive isolates (isolates 1–26) were initially identified as *Leuconostoc *by API 20 STREP. 7 catalase-negative gram-positive strains with vancomycin MIC ≥ 256 μg/ml were isolated during 2005–2006 (isolates 31–33 and 35–38). Thus, 33 clinical isolates entered the study. As 16S rDNA sequencing analysis was performed after the other tests, the results were not complete. Some isolates could not be retrieved, and some could not be amplified.

11 isolates of isolates 1–26 were reproducibly identified by API 20 STREP as *Leuconostoc *(Table 1). Only 3 of the 11 isolates, however, were confirmed by both genotypic and all defined phenotypic criteria (Table 1). 7 catalase-negative gram-positive isolates with vancomycin MIC ≥ 256 μg/ml (isolates 31–33 and 35–38) were all identified as *Leuconostoc *by API 20 STREP, only 2 of which were confirmed genotypically. API 20 STREP identified *Lactobacillus pentosus *ATCC 8041 and *Pediococcus pentosaceus *ATCC 33316 as *Leuconostoc *with 81.1% and 39.3% identity, respectively. Regarding all 31 non-leuconostoc strains (including reference strains and clinical isolates), API 20 STREP identified 10 of them as *Leuconostoc *with over 90% identity. 16S rDNA sequencing data were available in 7 of the 10 isolates and all were closely-related *Weissella spp*. 6 isolates were read as *Leuconostoc *with 50–90% identity. Two of these were *Lactobacillus pentosus *ATCC 8041 and *Lactobacillus salivarius *(isolate 26) and two were *Pediococcus *(isolates 35 and 38). API 50 CHL identified almost all *Leuconostoc *correctly, at least to the genus level, except for isolate 3. The kit, however, identified *Pediococcus pentosaceus *ATCC 33316, 7 out of 8 *Weissella spp*., all 6 streptococci, and 1 *Enterococcus *as either *Lactobacillus *or *Leuconostoc*. Of all 37 standard and clinical strains, 14 demonstrated at least one discrepant biochemical test results between API 20 STREP and manual phenotypic assays (Table 1). All these belonged to non-leuconostoc isolates and do not affect conventional phenotypic interpretation of these isolates as non-leuconostoc. Identity percentages given by API 20 STREP and API 50 CHL had poor correlations with PCR or phenotypes.

With regard to the 7 isolates of catalase-negative gram-positive bacteria with high-level vancomycin resistance which were all identified as *Leuconostoc *by API 20 STREP, only 2 of them were confirmed as such by genotypic assays. API 50 CHL correctly identified both isolates as *Leuconostoc*, one of which was correct at the species level.

## Discussion

Commercial diagnostics have been widely used in bacteriology laboratories to identify common organisms, such as *Streptococcus*, to the species level, or to identify unusual gram-positive organisms in clinical specimens, e.g. *Aerococcus*, *Lactobacillus*, and *Leuconostoc*, among others. Studies illustrating inaccurate identification of various gram-positive pathogens have been published [3,10-14]. In this study, our purpose is to raise an awareness that *Leuconostoc*, an emerging human pathogen, can be overdiagnosed by certain commercial diagnostics.

Occasional discrepant results among the same biochemical tests obtained from API 20 STREP and from manual conventional assays are not unexpected, as incomplete agreement of various automated and manual systems have been reported [15-17]. Reproducibility of API 20 STREP for *Leuconostoc *identification is only moderate in our study. Previous studies have shown higher consistency of bacterial identification by commercial diagnostics [18,19]. Clinical isolates in our study were initially identified during the time spanning from 1997–2006 and thus repeated API 20 STREP testing was done months or years thereafter. Our lower reproducibility could be, at least partly, due to loss or change in some characteristics by repeated subculture [20,21].

All 6 standard and clinical *Leuconostoc *strains were correctly identified by API 20 STREP and 5 by API 50 CHL, at least at the genus level. On the contrary, specificity of *Leuconostoc *identification by these API kits were only moderate at best. As evidenced by 16S rDNA sequence analysis, most of the isolates misidentified as *Leuconostoc *by API systems were in the genus *Lactobacillus *and *Weissella*, which are closely-related bacteria, followed by *Pediococcus*, also one of the lactic acid bacteria [6]. API 20 STREP failed to identify these isolates obviously because *Lactobacillus *and *Weissella *are not listed in the Identification Table [22]. Even some strains of streptococci and enterococci initially were identified as Leuconostoc. *Streptococcus constellatus *isolate 19 was misidentified at the species level. API 50 CHL misidentified most *Weissella *in this study obviously because only one species, *Weissella viridescens*, is included in the Identification Table of the kit.

Our conventional phenotypic criteria correlated well with *Leuconostoc*-specific PCR and 16S rDNA sequence analysis in almost all isolates, except for isolates 7 and 36 (Table 1). Isolate 7 was phenotypically compatible with *Leuconostoc *but negative by PCR. This could be closely-related bacteria which are certain lactobacilli such as *L. sanfrancisco*, or *L. fructosus*, or *Weissella *[23], or some rare *Leuconostoc *not detected by our PCR protocol. *Weisella *is a recently-described genus found in a variety of foods. Some of its members used to be *Leuconostoc paramesenteroides *and heterofermentative lactobacilli. Reliability of the conventional phenotypic criteria in this study is evidenced by the fact that only 1 of the 8 *Weissella *isolates and none of the 2 *Lactobacillus *isolates (one identified by 16S rDNA sequencing and both by PCR) was misidentified as *Leuconostoc*.

The importance of accurate identification of *Leuconostoc *also needs to be emphasized in the clinical arena. Case reports based on incomplete and/or inappropriate phenotypic criteria with or without assistance of commercial diagnostics are subject to potential errors [24-27], given the fact that *Leuconostoc *and related bacteria possess overlapping phenotypes. Flawed clinical reports include an incorrect argument that heterofermentative *Lactobacillus *must hydrolyze arginine [25], while in fact *L. sanfrancisco *and *L. fructosus *do not [23], and labeling the organism as *Leuconostoc *even though the organism was LAP positive [26].

Two major limitations of API 20 STREP are noted. Firstly, the test contains *Leuconostoc *in its list, while some other medically-important lactic acid bacteria with overlapping phenotypes such as *Lactobacillus*, *Weissella *and *Pediococcus*, are not included. It is of note, however, that, according to the manufacturer, *Leuconostoc *is a multiple taxon of *Leuconostoc *and *Lactobacillus *and if a strain is identified as *Leuconostoc*, a note "POSSIBILITY OF *Lactobacillus *spp" is included in the report. Considering *Leuconostoc *as a multiple taxon of *Leuconostoc *and *Lactobacillus *by the manufacturer is not very practical, as *Leuconostoc *and *Lactobacillus *are distinct bacteria, microbiologically and clinically. Given that human infections by these lactic acid bacteria are emerging, these organisms could obviously be misidentified as *Leuconostoc *by API 20 STREP, potentially contributing to cumulative incorrect reporting in medical literature and incorrect understanding of its clinical spectra and epidemiology. Secondly, while clinical isolates of *Leuconostoc*, as a rule, are LAP and ADH negative, the test lists *Leuconostoc *as 70% LAP and 10% ADH positive [22]. Appropriate modifications of the kit criteria for *Leuconostoc *would significantly enhance its accuracy. For clinical laboratories, we propose that all catalase-negative gram-positive coccoid or coccobacillary bacteria with high level of vancomycin resistance (MIC ≥ 256 μg/ml) be tested with the manual phenotypic assays listed in Table 1: Gram's staining of the isolate grown in thioglycolate broth, arginine dihydrolase (ADH), leucine arylamidase (LAP), gas production in MRS broth, and pyrrolidonyl arylamidase (PYR) test. Users of this method are to accept that, even though more accurate than the current API systems, these conventional assays could still occasionally misidentify certain lactobacilli and *Weissella *as *Leuconostoc*. This practical guide should minimize avoidable inaccurate identification of this emerging pathogen.

## Conclusion

The current API systems, similar to some other commercial identification systems for microorganisms, still need improvement before they can reliably identify certain unusual gram-positive pathogens. They lack specificity in *Leuconostoc *identification. We propose that, for accuracy and reliability, identification of vancomycin-resistant, catalase-negative gram-positive bacteria be performed by practical, conventional phenotypic assays, with assistance of a genotypic confirmation where available.

## Competing interests

The author(s) declare that they have no competing interests.

## Authors' contributions

WK conceived of and designed the study, participated in its coordination, analyzed data, and drafted the manuscript. SN participated in coordination of the study, carried out experiments on API systems and conventional assays of bacteria, and assisted with PCR assays. TC performed 16S rDNA sequence analysis. SK carried out all PCR assays. CU performed susceptibility tests. AC helped design and coordinated the study. All authors read and approved the final manuscript.

## Pre-publication history

The pre-publication history for this paper can be accessed here:


